# Bone Mineral Density Loss in People With Epilepsy Taking Valproate as a Monotherapy: A Systematic Review and Meta-Analysis

**DOI:** 10.3389/fneur.2019.01171

**Published:** 2019-11-08

**Authors:** Rui Zhong, Qingling Chen, Xinyue Zhang, Mengmeng Li, Jianmin Liang, Weihong Lin

**Affiliations:** ^1^Department of Neurology, The First Hospital of Jilin University, Changchun, China; ^2^Department of Hepatology, The First Hospital of Jilin University, Changchun, China; ^3^Department of Pediatric Neurology, The First Hospital of Jilin University, Changchun, China

**Keywords:** bone mineral density loss, epilepsy, valproate, meta-analysis, duration of VPA therapy

## Abstract

**Objective:** Data on changes in bone mineral density (BMD) from valproate (VPA) therapy are ambiguous and conflicting. Thus, the aim of this study was to systematically review the existing data and carry out a meta-analysis to investigate the effect of VPA as a monotherapy on BMD in people with epilepsy (PWE).

**Methods:** We systematically searched PubMed, EMBASE, and MEDLINE for eligible studies. We calculated the standardized mean difference (SMD) with 95% confidence interval (CI) to investigate the statistical power of the association between VPA treatment and BMD.

**Results:** Nineteen studies were included in this systematic review and meta-analysis. We found that BMD was lower in the VPA group than in the control group (SMD: −0.44; 95% CI: −0.65 to −0.22). A significant association was found in adult patients (SMD: −0.57; 95% CI: −0.88 to −0.26; *I*^2^ = 69.8%) and pediatric patients (SMD: −0.32; 95% CI: −0.60 to −0.03; *I*^2^ = 67.8%) by subgroup analysis. This study indicated that BMD was significantly lower in patients treated for more than 36 months than in controls (SMD: −0.52; 95% CI: −0.76 to −0.27; *I*^2^ = 61.8%). However, a significant difference was not found between patients who were treated for less than 36 months and controls (SMD: −0.36; 95% CI: −0.72 to 0.01; *I*^2^ = 74.8%).

**Conclusion and significance:** The present study provided evidence that VPA treatment was significantly associated with BMD loss in PWE. Thus, for patients at a high risk of osteoporosis and fracture, especially for patients who need long-term treatment, VPA may not be a good choice.

## Introduction

Epilepsy is one of the most common neurological disorders and is characterized by chronic and spontaneous epileptic seizures (1, 2). It was estimated that more than 50 million patients are affected by epilepsy worldwide (3). Antiepileptic drugs (AEDs) are first-line treatments for people with epilepsy (PWE) and are able to control epileptic seizures in approximately 60–70% of patients (4). However, it has been reported for years that long-term AED therapy may cause adverse effects in the bone and vitamin D deficiency (5–8). The relationship between epilepsy, AEDs, and bone health has been explored for more than three decades (5–9). Evidence from previous studies demonstrated that old AEDs such as carbamazepine (CBZ) had large effects on bone mineral density (BMD) and the vitamin D status (10). However, for PWE taking new AEDs, the abnormalities in vitamin D, calcium and BMD tended to be less severe (11, 12).

AED therapy could lead to bone disease and could increase the risk of fracture (13). However, the specific association of the type of AED with bone disease and fracture risk remains uncertain. Old enzyme-inducing AEDs (EIAEDs), such as CBZ, are the most commonly associated AEDs with bone metabolism abnormalities. Cytochrome P450 (CYP450) is induced by CBZ and may lead to bone metabolism abnormalities, which is reflected by an increased concentration of the biochemical markers of bone resorption and degradation in both serum and urine (14). Valproate (VPA) is an inhibitor of the CYP450 enzyme and may have minimal effects on hepatic metabolic enzymes. However, the effect of VPA on the bone is still controversial.

A great amount of attention has been paid to the association between VPA treatment and bone health over a number of years. Recent progress revealed that VPA treatment could lead to a decrease in vitamin D levels in pediatric patients with epilepsy, which partly explained the adverse bone-related side effects of VPA therapy (15). Vitamin D deficiency was found to be an important risk factor that could lower both lumbar and femur BMD (16). However, data on changes in BMD from VPA are still ambiguous and conflicting. Significant BMD loss was found in patients treated with VPA for more than 6 months by Pitetzis et al. (17). Thus, high-level evidence from a systematic review and meta-analysis was required to determine how VPA monotherapy affected BMD in PWE. The objective of this study was to systematically review the existing evidence and to carry out a meta-analysis to investigate the effect of VPA monotherapy on BMD in PWE.

## Methods

This systematic review and meta-analysis was performed in accordance to the Preferred Reporting Items for Systematic Reviews and Meta-Analyses guidelines (18).

### Search Strategy

Two independent reviewers (Zhong and Chen) systematically searched PubMed, EMBASE, and MEDLINE from inception to July 1, 2009, for eligible studies. All published studies investigating the effect of VPA on BMD were included in this review. The following search terms were used: “Valproic acid” OR “valproate” OR “VPA” AND “epilepsy” OR “epileptic” OR “seizure” AND “bone mineral density” OR “bone density” OR “BMD.” The medical subject headings (MeSH) search and free-text terms search were performed with our search strategy. The published language of articles was limited to English. The reference lists of relevant studies and reviews were also screened for additional eligible studies. Disagreements were discussed and resolved by consensus.

### Study Selection

Studies were included in the present study if they met the following inclusion criteria: (1) the study was a cross-sectional study, case–control study or prospective observational study. (2) The participants in the observation group were PWE taking VPA as a monotherapy. (3) Comparisons were performed with healthy controls or PWE who were not receiving AEDs. (4) BMD was reported as an outcome. All studies investigating the effect of VPA on BMD in patients with epilepsy were included in the current review if they met these inclusion criteria. The exclusion criteria were as follows: (1) case reports, reviews, editorials, comments, abstracts, or meta-analysis articles; (2) molecular biology or animal research; and (3) studies with duplicated or overlapping data. The titles and abstracts of each study were evaluated after removing duplicates. Then, the studies were assessed for eligibility by examining the full text and using the specified inclusion criteria. The study selection process was carried out by two independent reviewers (Zhong and Chen). Disagreements were discussed and resolved by consensus.

### Data Extraction

Each included study was screened, and data were extracted by two independent reviewers. The following data were extracted: first author, publication data, country, sample size, age group, VPA duration, and study design. Additionally, detailed information on BMD was required to combine the results. Disagreements between the two reviewers (Zhong and Chen) regarding data extraction were resolved through face-to-face discussions.

### Quality Assessment

The Newcastle-Ottawa scale (NOS) was used to assess the quality of each included study (19). The scores ranged from 0 stars (worst) to 10 stars (best). Studies with a score of 6 stars or higher were classified as high quality, and those with scores of less than 6 stars were classified as poor quality. Two reviewers (Zhong and Chen) independently evaluated the quality of the included studies, and disagreements were resolved by discussion.

### Statistical Analysis

We calculated the standardized mean difference (SMD) with 95% confidence intervals (CIs) to investigate the statistical power of the association between VPA treatment and BMD. The results are presented as forest plots, which included the contribution of each study (weight) to the overall effect. The heterogeneity was assessed by the *P*-value of the X^2^ and *I*^2^ statistics, and the heterogeneity was significant if the *I*^2^ statistic was greater than 50% or if the *P*-value was less than 0.05. We pooled data with a random-effects model if the *I*^2^ statistic was greater than 50%. Otherwise, a fixed-effects model was used. Childhood and adolescence are the most important periods for bone development. Thus, the effect of VPA on bone metabolism may be associated with age. Thus, a subgroup analysis based on age was conducted. Additionally, a subgroup analysis on the duration of VPA was also performed. Sensitivity analysis was used to examine the stability of the results by sequentially removing each study. Publication biases were testified by a funnel plot and Egger's test. All statistical analyses were conducted with STATA 12.0 software. A *P*-value of < 0.05 was considered statistically significant.

## Results

### Study Selection

**Figure 1:**
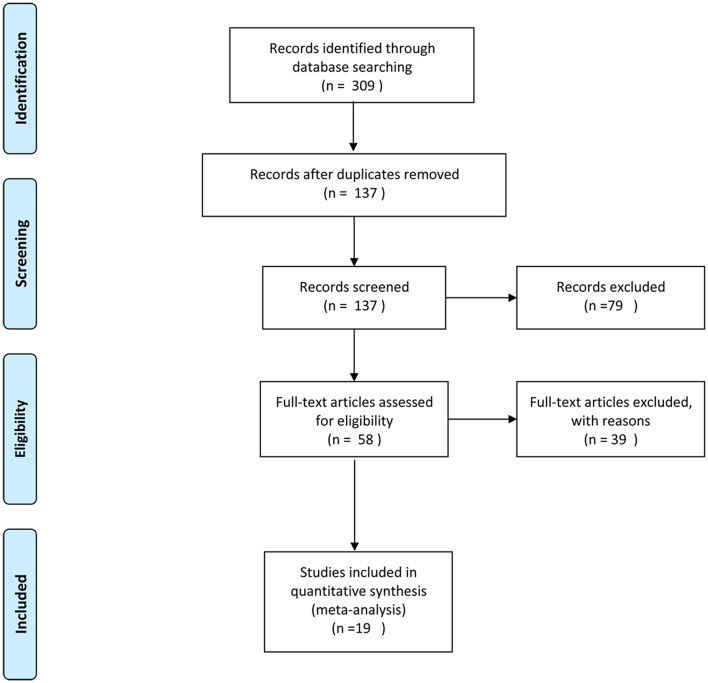
Flowchart of the study identification, inclusion, and exclusion in this systematic review.

A total of 451 articles were identified with the search strategy. The title and abstract of each article were screened after the exclusion of duplicates. Then, 69 articles were assessed for eligibility using the specified inclusion criteria by examining the full text. A total of 19 studies (6–36) met the inclusion criteria for qualitative synthesis. A flow diagram of the study selection is shown in Figure 1.

### Study Characteristics

Out of 19 observational studies (including 601 participants in the observation group and 671 controls) identified from the systematic review, nine were conducted in Turkey, two in Iran, two in Korea, and one each in Iraq, Syria, Australia, Edirne, USA, and Japan. The sample sizes among these studies ranged from 28 to 103. The mean duration of VPA therapy ranged from 4 to 116 months. The BMD values of the patients who took VPA for more than 36 months were reported by 10 studies. The BMD values of patients who took VPA for no more than 36 months were reported by nine studies. Nine of the included studies were performed in adult patients, and 10 were performed in pediatric patients. There were 14 cross-sectional studies, 3 prospective studies, and 1 case–control study in this meta-analysis. The mean NOS score was 6.74 (ranging from 5 to 8), indicating that the included studies were high quality. The characteristics of the included studies are summarized in Table 1.

**Table 1:** Basic characteristics of the include studies in the review.

**References**	**Country**	**Age (years)**		**Gender** **(male/female)**		**Sample size**		**Age group**	**VPA duration (months)**	**Study design**	**Diagnosis**
		**VPA**	**Controls**	**VPA**	**Controls**	**VPA**	**Controls**				
Kamil et al. (29)	Iraq	20.1	29.1	18/22	18/22	40	40	Adults	59	Case–control	Active epilepsy
Albaghdadi et al. (32)	Syria	26	27.2	17/33	18/32	50	50	Adults	100	Cross-sectional	Epilepsy
Shiek Ahmad et al. (23)	Australia	43	44	7/6	33/20	13	53	Adults	4	Cross-sectional	Epilepsy
Serin et al. (26)	Turkey	8.11	7.6	17/11	7/13	28	20	Children	24	Cross-sectional	Epilepsy
Yaghini et al. (33)	Iran	7.4	7.9	NR	NR	30	30	Children	6	Cross-sectional	Epilepsy
Salimipour et al. (27)	Iran	21.8	30	10/12	8/30	22	38	Adults	43	Cross-sectional	Epilepsy
Aksoy et al. (10)	Turkey	8.42	8.16	28//25	19//31	53	50	Children	39	Cross-sectional	Idiopathic generalized epilepsy
Heo et al. (28)	Korea	28.5	28.4	32/0	36/0	32	36	Adults	79	Cross-sectional	Epilepsy
Musa et al. (30)	Edirne	6.77	7.77	35/26	35/26	61	61	Children	12	Prospective	Epilepsy or complex febrile seizure
Pack et al. (20)	USA	30	30	NR	NR	14	14	Adults	48	Prospective	Epilepsy
Kim et al. (35)	Korea	26	26	10/5	10/5	15	15	Adults	6	Prospective	Newly diagnosed epilepsy
Babayigit et al. (21)	Turkey	11.18	13.09	15/16	14/16	31	30	Children	40	Cross-sectional	Primary idiopathic epilepsy
Kumandas et al. (34)	Turkey	8.8	8.9	17/16	13/9	33	22	Children	34	Cross-sectional	Epilepsy
Boluk et al. (22)	Turkey	30	30	24/26	30/30	50	60	Adults	80	Cross-sectional	Epilepsy
Ecevit et al. (24)	Turkey	10.59	11.52	6/10	17/14	16	31	Children	32	Cross-sectional	Idiopathic epilepsy
Öner et al. (31)	Turkey	7.1	7.4	17/16	17/16	33	33	Children	6	Cross-sectional	Epilepsy
Sato et al. (36)	Japan	37.4	35.3	23/17	22/18	40	40	Adults	116	Cross-sectional	Epilepsy
Erbayat Altay et al. (25)	Turkey	10.9	10.65	10/5	14/8	15	22	Children	37	Cross-sectional	Idiopathic general seized epilepsy
Akin et al. (6)	Turkey	8.8	8.9	14/11	15/12	25	26	Children	29	Cross-sectional	Primary epilepsy

*VPA, valproate; NR, no reported*.

### Meta-Analysis

**Figure 2:**
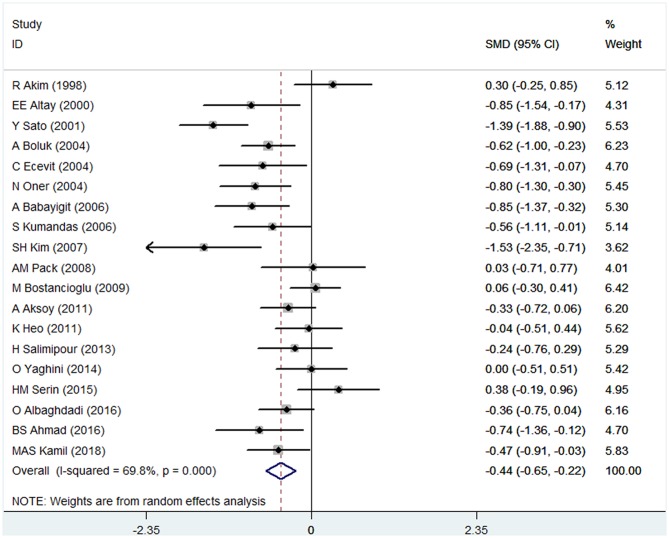
Forest plot of BMD in the VPA group and the control group. The size of the square reflects the study's weight. Each horizontal line represents the 95% CI of the SMD. The diamond represents the pooled SMD. SMD, standardized mean difference; CI, confidence interval.

There were 19 studies (with 1272 individuals) included in the data analysis. The pooled SMD data indicated that a lower BMD was observed in the VPA group than in the control group (SMD: −0.44; 95% CI: −0.65 to −0.22) (shown in Figure 2). There was significant heterogeneity across studies (*I*^2^ = 69.8%). Thus, a random-effects model was used.

### Subgroup Analysis

Subgroup analysis was conducted for the various age groups. The combined results showed that compared to the controls, both adult patients taking VPA (SMD: −0.57; 95% CI: −0.88 to −0.26; *I*^2^ = 69.8%) and pediatric patients taking VPA (SMD: −0.32; 95% CI: −0.60 to −0.03; *I*^2^ = 67.8%) had low BMD in subgroup analysis.

Subgroup analysis was also conducted based on the duration of VPA treatment. Studies were divided into two groups: studies with a mean duration of VPA therapy ≥36 months and studies with a mean duration of VPA therapy <36 months. The pooled SMD indicated that BMD was significantly lower in patients who took VPA for more than 36 months than in controls (SMD: −0.52; 95% CI: −0.76 to −0.27; *I*^2^ = 61.8%). However, there was no significant difference in the BMD value of patients who took VPA for less than 36 months and controls (SMD: −0.36; 95% CI: −0.72 to 0.01; *I*^2^ = 74.8%).

### Sensitivity Analysis

**Figure 3:**
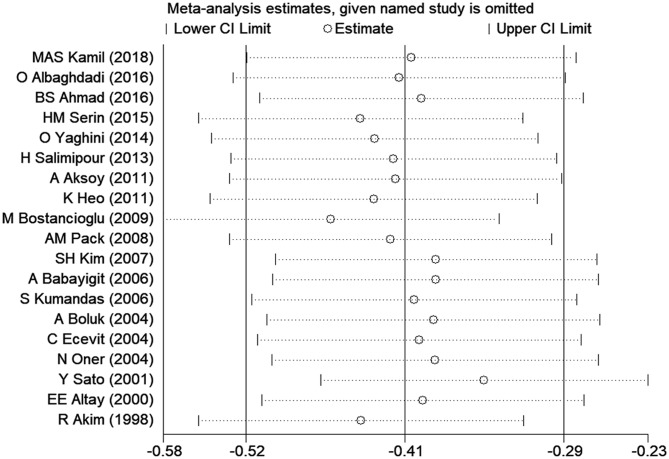
Sensitivity analysis indicated the robustness of the results. CI, confidence interval.

Sensitivity analysis was carried out to assess the impact of each individual study on the overall SMD. The stability and reliability of the overall SMD were examined by removing one study at a time (shown in Figure 3). We found that the results were generally constant and stable.

### Publication Bias

**Figure 4:**
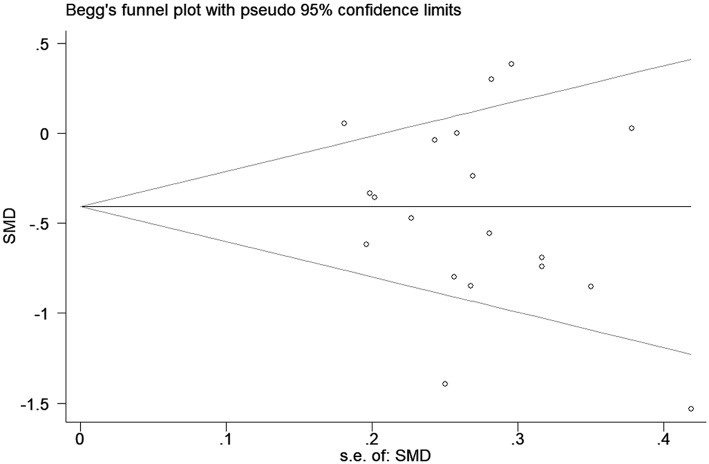
Funnel plot of the selected studies. These results suggest publication bias. SMD, standardized mean difference.

Publication biases were investigated by sequentially removing each study. There was no obvious publication bias based on the funnel plots (shown in Figure 4). This result was also supported by Egger's test (*P* = 0.529).

## Discussion

In this meta-analysis of 19 studies, we found a significant association between low BMD and long-term VPA therapy in PWE. In other words, VPA treatment could increase the risk of BMD loss in PWE. In addition, the adverse effect of VPA on BMD was observed in both adult patients and pediatric patients. We found that BMD was associated with the duration of VPA treatment. The results showed a low BMD in PWE who were treated with VPA for more than 36 months. No significant difference was found in the BMD value between controls and patients who took VPA for less than 36 months. This systematic review and meta-analysis provides sufficient data to support the adverse effect of VPA on BMD in PWE.

VPA has been approved as a first-line AED for idiopathic and symptomatic generalized epilepsy for more than three decades due to its obvious anticonvulsive function (17). However, the adverse effects of VPA treatment should not be ignored. Vitamin D deficiency, osteoporosis, bone loss, and an increased risk of fracture have been reported as potential adverse effects on the bone of long-term VPA therapy (36–38). However, there were inconsistent results among studies. A recently published meta-analysis provided evidence that long-term VPA therapy in children with epilepsy was significantly associated with vitamin D deficiency. A significant decrease in vitamin D levels was observed in children with epilepsy receiving VPA monotherapy compared with healthy children (15). The important role of vitamin D in bone mineral metabolism is well known. In addition, a study by Cheng et al. aimed to investigate the risk of fracture in old patients who were treated with different AEDs. These researchers found that VPA treatment was not associated with an increased fracture risk in elderly patients (39). Low BMD was significantly associated with an increased risk of fractures in patients who were treated with VPA. However, the evidence regarding the effect of VPA therapy on BMD was still ambiguous. RD Sheth et al. first reported that VPA treatment could reduce BMD in children with epilepsy, which may have been associated with an increased risk of osteoporotic fractures in these patients (40). Similar results were obtained by some studies (33–36). Additionally, several subsequent studies failed to find an association between BMD and VPA treatment in patients (25–28). The inconsistent results may be partly explained by differences in the basic characteristics among these studies. The adverse effect of VPA on the bone may be associated with the duration of treatment. Pitetzis et al. systematically reviewed related studies and found that there was significant BMD loss in patients treated with VPA for more than 6 months (17). This finding was consistent, to some extent, with the results of our subgroup analysis. Additionally, this result was limited by being obtained from a small simple size in a single study. This meta-analysis of 19 studies has sufficient statistical power to come to a reliable conclusion. Aiming to evaluate the effect of VPA therapy on BMD in patients with epilepsy, we systematically reviewed the evidence and pooled the data of published studies that focused on the association between BMD and VPA treatment. Our study found that patients with epilepsy who received VPA therapy had a significantly low BMD. The adverse effects of VPA treatment on BMD were found in both adult patients and pediatric patients in the subgroup analysis. In addition, we also found that BMD loss was significantly associated with the duration of VPA treatment in patients. BMD was lower in patients treated with VPA for more than 36 months than in controls. However, a significant association was not observed in patients who took VPA for less than 36 months.

A published meta-analysis indicated that VPA treatment may have been associated with decreased BMD in children with epilepsy (41). However, there were several differences between our meta-analysis and that published study. First, that published review included five studies. More studies on this topic were performed in recent years, and the current review included 19 studies, which provided increased statistical power. Second, the published study aimed to assess the effect of VPA on BMD in pediatric patients. The present study aimed to investigate the effect of VPA therapy on BMD in PWE. The association between BMD and VPA treatment was further analyzed in both adult patients and pediatric patients in a subgroup analysis. Third, subgroup analysis based on the duration of VPA treatment was also performed to assess the association between BMD and the duration of VPA therapy. Thus, the present study more accurately and comprehensively described the effect of VPA on BMD. Additionally, more findings could be obtained from the present meta-analysis.

Although the exact mechanism for the significant association between VPA treatment in epilepsy and BMD loss remains unclear, several theories have been proposed to explain this adverse effect of VPA treatment. A study by Fuller et al. revealed that VPA therapy was associated with a reduction in the production of two important bone proteins, collagen I, and osteonectin, which may explain the BMD loss after long-term VPA treatment (42). A subsequent study also demonstrated that the levels of pro-collagen I and osteonectin were significantly and substantially decreased after 24 h of exposure to VPA (43). Collagen I is the main protein component of the bone matrix, and osteonectin plays a key role in the development of bone. Thus, reduced levels of pro-collagen I and osteonectin may lead to low BMD in response to long-term exposure to VPA.

Exposure to VPA may be associated with cell morphology changes in osteoblast and fibroblast cell lines, indicating that VPA may rearrange the cytoskeleton of various cell types (44, 45).

Several limitations in this systematic review and meta-analysis should be acknowledged. First, significant, high heterogeneity was observed in most analyses, indicating that there were some confounding variables. We explored the potential source of heterogeneity by performing subgroup analysis and sensitivity analysis. However, the source of heterogeneity was not identified. Differences in inclusion criteria and basic characteristics among studies may have led to heterogeneity. Second, women and the elderly are more prone to bone problems. However, it remains unclear whether there is a sex difference in the effect of VPA on BMD. Subgroup analysis based on sex was not performed due to a lack of detailed information. This study also could not examine the effect of VPA on BMD in elderly patients with epilepsy. Third, only articles published in English were included in our meta-analysis. A meta-analysis may be biased when the literature search fails to identify all relevant studies. However, access to unpublished articles remains difficult, which might be a potential limitation of our study.

## Conclusion

In summary, the present study provided evidence that VPA treatment was significantly associated with BMD loss in PWE. Significantly low BMD was observed in both adults and children treated with VPA. We also found that the effect of VPA on BMD was associated with the duration of VPA treatment. Low BMD was observed in patients taking VPA for more than 36 months. Thus, for PWE who are at a high risk of osteoporosis and fracture, especially for patients who need long-term VPA treatment, VPA may not be a good choice. Additional well-designed studies with a large sample size are required to prove our findings.

## Author Contributions

RZ and QC designed the study, reviewed the literature, conducted the statistical analysis, drafted the manuscript, and discussed the manuscript. ML generated summary tables and edited pictures. XZ and WL significantly contributed to the study design. JL contributed to the embellishment of language and revision of the manuscript.

### Conflict of Interest

The authors declare that the research was conducted in the absence of any commercial or financial relationships that could be construed as a potential conflict of interest.
